# Do ancient types of wheat have health benefits compared with modern bread wheat?

**DOI:** 10.1016/j.jcs.2017.11.010

**Published:** 2018-01

**Authors:** Peter R. Shewry

**Affiliations:** Department of Plant Science, Rothamsted Research, Harpenden, Hertfordshire, AL5 2JQ, UK; School of Agriculture, Policy and Development, University of Reading, Earley Gate, Reading RG6 6AR, UK

**Keywords:** Ancient wheat, Health benefits, Allergy, Intolerance

## Abstract

A number of studies have suggested that ancient wheats have health benefits compared with modern bread wheat. However, the mechanisms are unclear and limited numbers of genotypes have been studied, with a particular focus on Kamut^®^ (Khorasan wheat). This is important because published analyses have shown wide variation in composition between genotypes, with further effects of growth conditions. The present article therefore critically reviews published comparisons of the health benefits of ancient and modern wheats, in relation to the selection and growth of the lines, including dietary interventions and comparisons of adverse effects (allergy, intolerance, sensitivity). It is concluded that further studies are urgently required, particularly from a wider range of research groups, but also on a wider range of genotypes of ancient and modern wheat species. Furthermore, although most published studies have made efforts to ensure the comparability of material in terms of growth conditions and processing, it is essential that these are standardised in future studies and this should perhaps be a condition of publication.

## Highlights

• Critically appraises the health benefits of ancient wheats in relationship to grain composition.
• Includes human intervention trials and in vitro studies.
• Included metabolic and adverse effects.
• Makes recommendations for future work.

## 1 Introduction

Wheat is the dominant crop and major staple food in temperate countries, with the mean global production over the period 2010 to 2014 being about 690 million tonnes (http://www.fao.org/faostat/en/#data). It contributes between 20% and 50% of the total calories in wheat-producing countries but the consumption of wheat is also increasing in countries where it is not climatically adapted, including parts of Sub-Saharan Africa, and particularly in countries undergoing urbanisation (Mattei et al., 2015). Although wheat is often regarded mainly as a source of calories, it also contributes essential amino acids, minerals and vitamins, beneficial phytochemicals and dietary fibre components to the human diet (NDND, 2014, Shewry and Hey, 2015a). However, wheat products are also at the centre of concerns about the relationship between the western diet and lifestyle and health outcomes, and particularly the increasing prevalences of obesity, type 2 diabetes, allergy and food intolerances. These concerns have been propagated by the popular press and social media and have generally not been substantiated by detailed scientific review (Brouns et al., 2013, Shewry and Hey, 2016). Similarly, although it has also been suggested that modern bread wheat differs in its composition and health benefits from traditional types of wheat (Morris and Sands, 2003), such differences have not been identified by detailed analyses (Shewry et al., 2011, Ribeiro et al., 2016) with the exception of a decreased content of mineral micronutrients (reviewed by Shewry et al., 2016, Shewry et al., 2017).

The concerns about the consumption of bread wheat have been accompanied by the promotion and increased consumption of ancient forms of wheat, based on perceived health benefits. However, genotypes of wheat vary widely in composition while ancient wheats may be grown and processed differently to modern bread wheats. It is therefore necessary to consider whether effects observed relate to intrinsic differences between wheat species or to variation between genotypes or to the impacts of differences in cultivation and processing.

### 1.1 What are ancient wheats?

Wheat was first cultivated about 10,000 years ago, as part of the “Neolithic Revolution”, which saw the transition from hunting and gathering of food to settled agriculture. The earliest cultivated forms were einkorn and emmer, which are diploid (genome AA) and tetraploid (genomes AABB) species, respectively. Both species probably originated from the south-eastern part of Turkey (Dubcovsky and Dvorak, 2007) with emmer being derived from the spontaneous hybridization of the ancestor of einkorn with a related species of wild grass. Thus both species arose from the domestication of natural populations and wild wheats related to both still grow in the Middle East. Modern durum (pasta) wheats have developed from the same wild ancestor as emmer and both emmer and durum are now regarded as forms of the same species (*Triticum turgidum*). By contrast to einkorn and emmer, bread wheat has only existed in cultivation, having arisen about 9000 years ago by hybridization of cultivated emmer with wild “goat grass” (*Triticum tauschii*). Hence, it is a hexaploid species with three genomes (AABBDD) each comprising 7 pairs of chromosomes.

Crop domestication is associated with the selection of a range of genetic traits, which are called the “domestication syndrome”. In wheat these traits include a change from hulled forms, in which the glumes of the flower adhere tightly to the grain and are not removed by threshing, to free-threshing forms in which the naked grain is released on threshing. Consequently, whereas most forms of einkorn and emmer are hulled, bread wheat is free-threshing. However, hulled forms of bread wheat do occur and are termed “spelt”. Because the free-threshing character is controlled by mutations at only two genetic loci (Dubcovsky and Dvorak, 2007) bread wheat and spelt are regarded as forms of the same species (*Triticum aestivum*). Bread wheat and spelt are readily inter-bred, which has resulted in many modern types of spelt containing genetic material from bread wheat which has been incorporated to improve their performance.

Although bread and durum wheats together account for the vast majority of global wheat production, einkorn (*Triticum monococcum*), emmer and spelt (“ancient wheats”) continue to be produced in small amounts (mainly for traditional foods) and increases in production, particularly of spelt, have occurred in recent years to satisfy the increasing demand for the health food market. These hulled wheats are often together called “*farro*” in Italy.

A further type of “ancient” wheat, called Kamut^®^, has been actively promoted over the past two decades (Abdel-Aal et al., 1998). Kamut seed was originally obtained from Egypt in the late 1940s, described as “mummy wheat” from an Egyptian tomb (see Moshenska (2017) for a discussion of “mummy wheat”). However, it is more likely to have been purchased from a street trader (http://www.kamut.com/en/discover/the-story). It is known to be a genotype of Khorasan wheat, a form of *T turgidum* related to emmer and modern durum wheats. Kamut^®^ is a registered trademark of Kamut International Ltd and is only grown on certified organic farms. Similarly, ‘the tetraploid Italian wheat Graziella Ra^®^ is also purported to be derived from an Egyptian tomb (http://www.girolomoni.it/en/cat0_18828_18856-graziella-ra.php). Comparative analyses show that Graziella Ra and Kamut are related but distinct (Colomba and Gregorini, 2011, Colomba et al., 2012).

## 2 Factors affecting the composition of wheat grain

It is logical to expect that these different types of wheat exhibit genetically-determined differences in composition which may result in different impacts on diet and health. However, the composition of the grain is also affected by environmental factors, and the interactions of these with the genotype, and it is therefore necessary to briefly consider the relative effects of these factors.

### 2.1 Genetics

Bread wheat spread rapidly from the Middle East across temperate zones of the world, reaching China by 3000 years ago and being introduced into the New World in the 16th century and Australia in the late 18th century (Feldman, 2001). This migration was facilitated by the ability of wheat to adapt to local environments, resulting in vast genetic diversity in modern bread wheat. In 2001 Feldman (2001) noted the existence of 25,000 different cultivated forms of bread wheat and it is likely that the total current number is at least twice this estimate. These types not only differ in their adaptation to local environments, but are also likely to differ in their compositions, including their contents and compositions of “bioactive” components. Although large scale detailed comparisons are lacking, an indication of this diversity is given by the study initiated as part of the EU Healthgrain programme (2005–10). This included analyses of phenolics (phenolic acids, alkylresorcinols), terpenoids (sterols, stanols, tocols), folates and dietary fibre components in a collection of 150 bread wheat lines of diverse type, geographical origin and date of release. The concentrations of phytochemicals in wholemeal varied widely, by 3.6-fold for phenolic acids, 2.9-fold for tocols, 2.8-fold for alkylresorcinols, 2.4-fold for folates and 1.4-fold for sterols, with the content of arabinoxylan (the major dietary fibre fraction) in white flour also varying by over two-fold (Ward et al., 2008, Shewry et al., 2010). This is discussed in more detail by Shewry et al., 2013, Shewry et al., 2017.

### 2.2 Environment

Grain composition is affected by both the environment and agronomy, particularly the type and amount of nitrogen fertilisation. Increased nitrogen application leads to higher protein content (Shewry et al., 2013), but this is accompanied by effects on protein composition, with high protein grain containing higher proportions of gluten storage proteins and of gliadin proteins within this fraction (Godfrey et al., 2010). High protein grain also contains higher contents of free amino acids (Claus et al., 2006) while restricted sulphur availability results in reduced grain protein content but accumulation of free amino acids, particularly asparagine (Granvogel et al., 2007).

Grain composition is also affected by the weather conditions, especially the temperature and water availability during grain development. For example, comparison of a set of 26 genotypes grown in 4 or 6 environments showed positive correlations between the contents of phytochemicals and the mean temperature during grain development, with some components also showing negative correlations with total precipitation over the same period (Shewry et al., 2010). By contrast, the contents of the water-soluble arabinoxylan fibre in bran and white flour were both negatively correlated with temperature and positively correlated with precipitation (Shewry et al., 2010).

The availability of data for multiple sites also allowed the variation in composition between the samples to be apportioned between the effects of genetics and environment (Shewry et al., 2010). Whereas some components showed high heritability, with over half of the variation in amount being ascribed to genetic effects (arabinoxylan in flour, tocols, sterols and alkylresorcinols in wholemeal), other components had low heritability with the environment having a much greater impact than genetics (phenolic acids, B vitamins, betaine). Effects of genotype × environment interactions were also identified for some components, but these are still poorly understood and require more detailed studies.

### 2.3 Do ancient wheats differ from bread wheat in grain composition?

Shewry and Hey (2015b) compared data for ancient wheats with modern durum and bread wheats. However, to minimise effects of the environment they only considered studies in which modern and ancient wheats were grown together in field experiments. They concluded that ancient wheats differ little from modern wheat species in their contents of most bioactive components and may be lower in some components such as dietary fibre. However, there is clear agreement in the literature that einkorn, emmer and Khorasan (Kamut) wheat all have higher high contents of the carotenoid lutein than bread wheat, which is selected for white colour. Modern durum wheat is also rich in lutein due to selection for yellow colour.

Five lines each of emmer, einkorn and spelt were also included in the Healthgrain study, allowing comparison with 150 bread wheat and 10 durum wheat lines. Multivariate Principal Component analysis (PCA) of “bioactive phytochemicals” (phenolic acids, alkylresorcinols, tocols, sterols, folates, betaine, choline), dietary fibre components (Shewry et al., 2013) and polar metabolites (Shewry et al., 2017) showed only partial discrimination between the ancient and modern types of wheat. By contrast, Bodroza-Solarov et al (2014) discriminated between wholemeal flours of 7 bread wheats and 10 spelts by multivariate analyses based on GC-MS analysis of lipophilic components. A more recent study reported metabolomic profiling of finely milled whole grains of 77 accessions of emmer, einkorn and spelt grown in replicated randomised field trials on two sites for two years, including conventional and organic plots on one site (Righetti et al., 2016). Alkylresorcinols provided the best discrimination between species, being higher in spelt and emmer and differing in homologue composition between species while the phospholipids phosphatidyl choline and lysophosphatidyl choline were higher in einkorn. Ziegler et al. (2015) also reported that the amount and composition of alkylresorcinols could be used to discriminate between species of different ploidy (einkorn v emmer/durum v bread/spelt), but not between species of the same ploidy level. However, a comparison of the major FODMAPs (glucose, fructose, raffinose and fructans) in five cultivars each of bread wheat, spelt and durum wheat and two cultivars each of emmer and einkorn failed to show a clear differentiation between the species (Ziegler et al, 2016).

Hence, it can be concluded that although ancient and modern wheats differ to a limited extent in composition, these differences are likely to be confounded by the effects of the environment unless the lines are grown together in randomised field plots.

### 2.4 Implications for comparative studies

These genetic and environmental impacts on grain composition have two important implications for designing clinical trials and evaluating their outcomes. Firstly, it cannot be assumed that single genotypes of bread or ancient wheats are “typical” of the species (and therefore the results obtained with single genotypes cannot be applied to the whole species). One option to overcome the wide variation in composition which exists within species is to blend samples of different genotypes, to achieve an “average” composition. Secondly, samples used for comparative intervention studies should be grown under similar conditions, if possible in randomised replicated plots. Lines should also be adapted to the growth conditions, to avoid stress responses in plants grown outside their area of adaptation.

## 3 Do ancient wheats have health benefits compared with modern wheats?

Six trials reported comparisons of Kamut or related forms of Khorasan wheat with modern durum and/or bread wheats, measuring effects on parameters related to cardiovascular disease, glycaemic index, type 2 diabetes and irritable bowel syndrome. However, none of these studies compared Kamut wheat grown in identical conditions to the control wheats, presumably because the growth of Kamut is strictly controlled. As stated on the Kamut^®^ web site (http://www.kamut.com/en/discover/the-trademark): “The KAMUT^®^ trademark is a guarantee that the khorasan wheat bearing it is always the original, unmodified, unhybridized and non-GMO variety. KAMUT^®^ khorasan wheat is also always grown certified organic and meets high purity, nutrition and quality standards”.

Scazzina et al (2008) obtained wholemeal Kamut and bread wheat flours from a local (Italian) supermarket and hence nothing is known about the growth conditions of the crops or the identity of the control wheat (although it would be expected to be a blend of commercial cultivars). Tortillas prepared with 60% flour had significantly higher fibre (6.7% compared with 3.5%) and lower starch (44.3% compared with 48.6%) when made from Kamut than from bread wheat, but did not differ in glycaemic index in an intervention trial.

A series of four studies from the same research team compared Kamut with bread and durum wheats. The first of these studies (Sofi et al., 2013) described the sourcing of organically grown Kamut from Saskatoon (Canada) and organically grown mixtures of Italian durum and soft (bread) wheats, although details of the cultivars included in these mixtures and their growth conditions were not reported. The Kamut and durum wheats were milled using the same equipment to give semi-whole-wheat semolina and the Kamut and bread wheats similarly milled to give semi-whole-wheat flour. No significant differences in composition were found between the Kamut and control durum semolinas, but differences between the flours were reported. In particular, the Kamut flour had a significantly higher % amylose in starch (34.50% compared with 28.12%), higher protein content (16.36% compared with 13.98%) and higher contents of “antioxidant phytochemicals “(polyphenols, carotenoids, flavonoids) and 2,2-diphenyl-1-picrylhydrazyl (DPPH) antiradical activity. The Kamut semolina and flour also contained significantly higher levels of minerals, which could relate to the mineral availability on the growth sites as this strongly affects the mineral content of the grain.

Pasta made from the semi-whole wheat semolina fractions of Kamut and durum wheat and bread and crackers made from the semi-whole wheat flours from Kamut and bread wheats were compared in a randomised single blinded cross-over trial with 22 patients. The Kamut diet resulted in significant reductions in metabolic risk factors (total cholesterol, LDL cholesterol, blood glucose), improved redox status, increased serum potassium and magnesium and significant reductions in circulating levels of pro-inflammatory cytokines.

A similar approach, comparing organic Kamut grown in Canada with mixtures of organically grown Italian durum and bread wheats, was used by the same authors to determine effects on irritable bowel syndrome (IBS) (Sofi et al., 2014), although in this case the semi-whole wheat semolina and flour from Kamut showed significantly higher levels of minerals, polyphenols and carotenoids than in the corresponding fractions of modern wheats. However, fermentable oligosaccharides, including fructans, were not measured. A double-blinded randomised cross-over trial comparing products from Kamut and modern wheats (bread, biscuits, crackers, pasta) with 20 participants with moderate IBS showed amelioration of the severity of IBS symptoms with the Kamut diet only.

Two further papers from the same group used a similar approach, comparing organically grown Kamut with Italian durum and bread wheats (Whittaker et al., 2015. 2017), but also determined ash content as a measure of the content of bran in the milled semi-whole-wheat semolinas and flours. In the study of Whittaker et al. (2015) the contents of antioxidant phytochemicals (polyphenols, flavonoids, carotenoids), anti-radical power (APP) and DPPH anti-radical activity were higher in the Kamut flour than in the control wheat flour, while flavonoids were higher in the Khorasan semolina than in the durum semolina. The Khorasan wheat flour and semolina also contained higher contents of selenium, which is consistent with the generally accepted view that wheat grown in North American wheats have higher selenium contents than European wheat due the higher content in the soil (as discussed below). A randomised double blind crossover trial with 22 patients with acute coronary syndrome (ACS) showed reduced risk profiles for those on the Kamut diet, with reduced levels of blood glucose, insulin, total cholesterol and LDL cholesterol, improved redox status, and a significant reduction in the pro-inflammatory Tumor Necrosis Factor (TNF)-alpha. Finally, Whittaker et al. (2017) used similar grain fractions (although not identical based on the analyses presented) in a randomised double blind crossover trial with 21 patients with type 2 diabetes, showing an improved metabolic profile (reduced levels of blood glucose, insulin, total cholesterol and LDL cholesterol), reduced levels of reactive oxygen species, vascular endothelial growth factor and interleukin-Ira, and a significant increase in total antioxidant capacity.

The final dietary intervention paper compared the effects of wholemeal Kamut and durum wheat foods (baked goods and pasta) on the gut microbiota and faecal metabolome of two random groups of 15 healthy adults (Saa et al., 2014). No details of the grain samples or processing were provided, or of the compositions of the grains and products. The authors concluded that the Kamut diet was “mainly characterised by the release of short chain fatty acids and phenol compounds, as well as by a slight increase in health promoting mutualists of the gut microbiota in comparison with the whole durum wheat”, but considered that the “slight differences could still be considered to be relevant as the two wheat cultivars are botanically very close” A related study compared the fermentation of soluble dietary fibre fractions from a commercial sample of Kamut (presumably grown in North America) with two old and seven modern durum wheat varieties grown in Italy (Marotti et al., 2011), using probiotic strains of *Lactobacillus* and *Bifidobacterium.* They concluded that both Kamut and the modern cultivar Solex had good potential as substrates. However, it should be noted that the system used was highly simplified compared with the complex system in the colon.

These human intervention studies are complemented by a series of four papers by the group of Bordoni comparing the effects of Kamut and conventional bread and durum wheats in rats (Gianotti et al., 2011, Benedetti et al., 2012, Carnevali et al., 2014, Valli et al., 2016). These report benefits of Kamut in protecting against oxidative stress and anti-inflammatory properties, with one paper (Valli et al., 2016) also reporting benefits of Kamut compared with Italian Khorasan wheat.

It is notable that these studies all focused on Kamut rather than other types of ancient wheat. This reflects the promotion of the grain, with most of the publications cited above acknowledging support for their work from either Kamut Enterprises of Europe or Kamut International USA. All of the studies are well designed and carefully carried out, but the focus on Kamut does affect the comparability of the material used as the Kamut samples had been grown in North America rather than under the same European conditions as the control samples. This is particularly relevant because for Kamut^®^ grain to be marketed it must have a protein content between 12% and 18% and contain 400–800 ppb selenium (Valli et al., 2016). Wheat grain grown in North America is notably richer in selenium than grain grown in Europe, the average content being about 10-fold higher (reviewed by Hawkesford and Zhao, 2017). It is therefore not surprising that there was significant variation in the relative selenium contents reported for the Kamut and control flours used in the intervention diets: from 1.07-fold higher to 3.2 fold higher, (Sofi et al., 2013, Sofi et al., 2014, Whittaker et al., 2015, Whittaker et al., 2017), while the differences were much greater (10-fold or greater) between the Kamut and control samples used in the rat experiments (Gianotti et al., 2011, Benedetti et al., 2012, Carnevali et al., 2014, Valli et al., 2016). Similar differences can also be identified between the contents of other “bioactive” components in the Kamut and control samples, as expected in view of the strong effects of environmental factors on the contents of phytochemicals and minerals in wheat (Shewry et al., 2010). It is clearly not possible to dissect the relative contributions of genotype, environment and the interactions of these two types of factor on the differences between the compositions of Kamut and the control wheats (and the impact of these on health outcomes). However, the results of the single study in which Khorasan wheat grown in Italy was compared with North American Kamut (Valli et al., 2016) indicate that environment plays a significant role.

The striking feature of these studies is the very wide range of benefits reported for Kamut, particularly in relation to the rather modest differences between the compositions of the diets. Although selenium is of particular interest because it has an established role in protecting against oxidative stress (Brenneisen et al., 2005), none of the effects have so far been directly related to any individual components, or even to specific combinations of these. A recent review by Bordoni et al (2017) concluded that the health benefits demonstrated for Kamut were an example of “synergism among different components” but did not take into account the differences in provenance of the materials used for comparisons.

## 4 Are ancient wheats less likely to provoke adverse reactions?

Wheat has been reported to provoke a range of adverse reactions, which can be divided into three broad classes: IgE-mediated allergies, T-cell mediated intolerances (notably coeliac disease) and a range of less well-defined conditions which can broadly be called “wheat (or gluten) sensitivity” (Sapone et al., 2012). It is often suggested, or assumed, that these responses are greater with modern than with ancient wheats (Quinn, 1999), and a range of studies have been reported which explore this possibility. These studies have mainly focused on comparative studies of protein fractions with little information provided on the origin and properties of the raw material used. However, it can be argued that in this case the provenance is less important because there are limited effects of environment on grain protein composition (rather than protein content) (Shewry, 2007).

### 4.1 Coeliac disease

The role of wheat gluten proteins in triggering coeliac disease (CD) is well-established and thirty-one short peptide sequences in wheat gluten proteins, and related proteins in barley and rye, have been defined as being coeliac toxic: these are often referred to as coeliac “epitopes”. However, mapping is incomplete and the number of distinct epitopes is a matter of on-going discussion (Sollid et al., 2012). A number of studies have focused on comparing the presence of coeliac toxic epitopes in ancient and modern wheats, either by determination of T-cell responses or the identification of specific epitopes using monoclonal antibodies.

Molberg et al. (2005) used T-cells specific for stimulatory epitopes in α-gliadins (9 cell lines) and γ-gliadins (6 lines) to compare chymotryptic digests of guten fractions from diploid and tetraploid species, including modern durum wheats, but not bread wheat, showing differences in relation to genome constitution and among genotypes of the same species. Notably, the immunodominant αG-33mer fragment of α-gliadin was associated with the presence of the D genome while accessions of A genome diploids (*T. urartu* and *T. monococcum)* differed in the expression of γ-gliadin T-cell epitopes. They concluded that these differences could be exploited to select lines with low CD toxicity. Spaenij-Dekking et al. (2005) also used T cells specific for stimulatory epitopes in α-gliadin, γ-gliadin (2 lines) and LMW subunits of glutenin (2 lines) and HMW subunits of glutenin to compare peptic tryptic digests of flours of diploid, tetraploid and hexaploid wheats, again showing wide variation in the levels of T-cell stimulatory epitopes within species. More recently, Šuligoj et al. (2013) used intestinal gluten-specific T-cell lines from 13 patients to compare peptic tryptic digests of gliadin fractions from a range of diploid and tetraploid wheats and modern bread wheat, concluding that all species triggered responses. By contrast, Vincentini et al. (2009) showed that peptic tryptic digests of gliadins from some landraces of “farro” (*T. turgidum* ssp. *dicoccum*) resulted in only low responses with T-cells from four coeliac patients, suggesting low coeliac toxicity.

Several studies have used monoclonal antibodies to probe wheat protein fractions for coeliac-toxic sequences. Spaenij-Dekking et al. (2005) used monoclonal antibodies to epitopes present in α-gliadin (Glia-α9), γ-gliadin, and LMW and HMW subunit of glutenin to screen the same accessions as described above using competition assays and western blotting, which essentially confirmed the results obtained in the same study using T-cells. van den Broeck and colleagues used antibodies to two α-gliadin coeliac epitopes (Glia-α9 and Glia-α20) to screen landraces (50 accessions) and cultivars (36) of bread wheat (van den Broeck et al., 2010b) and 103 accessions of tetraploid wheat including cultivars, wild species and land races (van den Broeck et al., 2010a). Overall, the major Glia-α9 epitope was more abundant in the modern wheats and the minor Glia-α20 epitope in the land races, but some modern cultivars and land races had low contents of both epitopes (van den Broeck et al., 2010b). Two studies also used monoclonal antibodies raised to α-gliadin epitopes, DQ2-Glia-α1 and DQ2-Glia-α2, to compare Kamut and Graziella Ra tetraploid wheats with modern durums (Gregorini et al., 2009, Colomba and Gregorini, 2012), providing no evidence that the ancient types were less coeliac toxic.

Gell et al. (2015) used the R5 antibody to ω-gliadin and the α-gliadin specific G12 to compare wild diploid and tetraploid species of *Aegilops* with ancient and cultivated modern wheats. This showed some correlation between the reactivity with the R5 antibody and ploidy level in the *Triticum* species. This was supported by comparison of epitope distribution in the sequences of 613 gluten proteins in the UniProt database. Gelinas and McKinnon (2016) also used the same two antibodies, and concluded that cultivated tetraploid wheats (emmer, Kamut, durum) had less epitopes reactive to both antibodies than hexaploidy wheats (spelt, bread wheat). However, these results were not confirmed by a more extensive study using the same R5 monoclonal antibody to compare modern bread wheats (53), bread wheat land races (19), spelt varieties (20), modern durum wheats (15) and durum wheat land races (19) (Ribeiro et al., 2016). This showed wide variation in the abundances of coeliac epitopes among genotypes of individual species, by 10-fold in some cases. The reactivity was similar in modern bread wheat, modern durum wheat and durum wheat land races, but generally higher in land races of bread wheat and in spelt.

It is difficult to draw general conclusions from these studies except for the wide range of variation in reactions among genotypes of all species. The number of epitopes may also reflect the ploidy, for example, epitopes characteristic of the D genome are absent from diploid and tetraploid species which lack this genome. However, it should also be noted that these studies used only one to four antibodies which target only a small proportion of the total number of coeliac epitopes in gluten proteins.

A series of studies have focused on the coeliac toxicity of einkorn (*T. monococcum*), following a report that peptic tryptic digests of gliadins from this species were unable to agglutinate K562(S) cells, which was suggested to imply low coeliac toxicity (De Vincenzi et al., 1996). A subsequent study reported that a peptic tryptic digest of gliadins from *T. monococcum* was not toxic to intestinal mucosal cells in an *in vitro* culture system compared with a similar preparation from bread wheat (Pizzuti et al, 2006). A more detailed study was reported by Gianfrani et al. (2012), who compared preparations from two genotypes each of *T. monococcum* (ID331 and Monlis) and bread wheat using gliadin-specific T-cell lines and clones, and organ cultures from jejunal biopsies to determine effects on innate and adaptive immune responses using immunohistochemistry (Gianfrani et al., 2012). All four lines activated a T-cell response but the *T. monococcum* line ID331 differed in that it was unable to active the innate immune pathway. Monlis is unusual among *T. monococcum* lines in that it lacks ω-gliadins and Iacomino et al. (2016) subsequently showed that ω-gliadin from *T. monococcum* line ID331, and a 19 residue synthetic peptide corresponding to part of the ω-gliadin sequence (QSFPQQPQRPQPFFQQPEQ), were able to protect human CaCo-2 intestinal epithelial cells against damage by bread wheat gliadin. They also noted that the peptide sequence contained a region closely related to a decapeptide from ω-secalin (QQPQRPQQPF) which they had previously shown to provide similar protection (De Vita et al., 2012).

Despite the close similarity of these peptides to many sequences present in gluten proteins, the presence of an arginine residue (R) is unusual and a search of a manually curated database of 630 discrete unique full length gluten protein sequences (Bromilow et al., 2017) failed to find matches although the related peptide QQPQQPQQPF occurred widely (S. Bromilow, personal communication). It is therefore likely that the arginine residue is essential for the biological properties that have been described.

It has also been suggested that low immune-toxicity of *T. monococcum in vivo* may depend on gastro-intestinal digestion, as gliadins from *T. monococcum* and bread wheat showed similar effects on T-cells after partial peptic tryptic digestion, but that more extensive digestion with gastro-intestinal enzymes resulted in greater breakdown of *T. monococcum* gliadins and reduced immune stimulatory properties (Gianfrani et al., 2015).

The same research group also compared the toxic effects of gliadins from *T. monococcum*, farro (*T. dicoccum* landraces), spelt and bread wheat on K562(S) (human leukaemia) and Caco-2/TC7 (human colon adenocarcinoma) cell lines (Vincentini et al., 2006, Gazza et al., 2010), showing that tryptic peptic digests from farro and *T. monococcum* lines which did not lack ω-gliadins (such as ID331) were not cytotoxic.

### 4.2 Non-coeliac wheat sensitivity

Carnevali et al. (2014) compared the effects of feeding pasta from Kamut and modern pasta wheats on inflammation and oxidative status in rats, showing that whereas the modern pasta resulted in effects on the morphology of the duodenal mucosa, these were not observed with Kamut. The Kamut diet also provided antioxidant protection (as discussed above) while differences in faecal metabolites suggested the diets had different effects on the gut microflora. The authors suggest that these effects may relate to effects of wheat diets on non-coeliac wheat sensitivity, although they note that there are no scientific studies showing that Kamut is more readily tolerated. Valerii et al. (2015) also compared the effects of wheat protein fractions on cultured peripheral blood mononucleated cells from patients with non-coeliac wheat sensitivity, suggesting that they were activated to a greater extent by proteins from modern wheat cultivars then from Kamut.

### 4.3 Wheat allergy

Classical (IgE-mediated) allergy to the consumption of wheat is rather rare (Zuidmeer et al., 2008). Although it is sometimes suggested that people suffering from allergy to bread wheat are able to tolerate spelt or other ancient wheats, only few studies address allergic responses. Klockenbring et al. (2001) used pooled sera containing IgE antibodies to cereal flours and pollen to determine IgE, IgA, IgG1 and IgG4 reactivity to water-soluble protein fractions from one bread wheat line, two spelts and two bread wheat x spelt “hybrids”. Although the sample number was low, this did not demonstrate substantial differences between the lines. A more extensive study compared the sera of 73 patients found to be RAST positive for wheat (Vu et al., 2014). Of these, 63% showed higher IgE reactivity to water-soluble proteins from wheat, 30% higher reactivity to spelt proteins and 7% indifferent responses to both cereal protein fractions. Finally, Simonato et al. (2002) showed no differences in the binding of IgE antibodies to soluble and insoluble (gluten) protein fractions from bread wheat and Kamut. However, it should be noted that only the last of these studies compared gluten protein fractions, with the two more extensive studies comparing only soluble proteins.

## 5 Conclusions

Although these is an increasing volume of comparative studies of modern and ancient wheats, it should be noted that there are still relatively few research groups working in the area, and hence many of the studies, particularly the dietary interventions, use similar materials and lack independent confirmation. The majority of the intervention studies have also compared Kamut with modern wheats, and are limited by the inability to compare samples grown under different conditions. Hence, it is not possible to conclude that the effects reported related to genetically-determined differences in grain composition rather than the effects of environment (or interactions between genotype and environment).

By contrast, studies of “adverse effects” have been reported by a larger number of research groups and, because isolated protein fractions are used for comparison, are less confounded by potential effects of environment on grain composition. However, the outcomes of these studies are less consistent, ranging from clear differences between species to no differences at all. It is not possible to reconcile these studies and reach clear conclusions.

Hence, the major conclusion from this review is that further studies are urgently required, particularly from a wider range of research groups, but also on a wider range of genotypes of ancient and modern wheat species. A similar conclusion has been reached by a review article which was not available until after the conclusion of the literature review for this article (Dinu et al., 2018): that “given the limited number of human trials, it is not possible to definitely conclude that ancient wheat varieties are superior to all modern counterparts in reducing chronic disease”.

Although most published studies have made efforts to ensure the comparability of material in terms of growth conditions and processing, it is essential that these are standardised in future studies and this should perhaps be a condition of publication.

## References

[bib1] Abdel-Aal E.-S.M., Sosulski F.W., Hucl P. (1998). Origins, characteristics and potentials of ancient wheats. Cereal Foods World.

[bib2] Bodroza-Solarov M., Vujic D., Acanski M., Pezo L., Filipcev B., Mladenov N. (2014). Characterisation of the liposoluble fraction of common wheat *(Triticum aestivum) and spelt (Triticum aestivum* ssp. s*pelta*) flours using multivariate analysis. J. Sci. Food Agric..

[bib3] Benedetti S., Primaterra M., Tagliamonte M.C., Carnevali A., Gianottic A., Bordoni A., Canestrari F. (2012). Counteraction of oxidative damage in the rat liver by ancient grain (Kamut brand Khorasan wheat). Nutrition.

[bib4] Bordoni A., Danesi F., Di Nunzio M., Taccar A., Valli V. (2017). Ancient wheat and health: a legend or the reality: a review on KAMUT Khorasan wheat. Int. J. Food Sci. Nutr..

[bib5] Brenneisen P., Steinbrenner H., Sies H. (2005). Selenium, oxidative stress, and health aspects. Mol. Aspect. Med..

[bib6] Bromilow S., Gethins L.A., Bucklet M., Bromley M., Shewry P.R., Langridge J.I., Mills E.C.N. (2017). A curated gluten protein sequence database to support development of proteomics methods for determination of gluten in gluten-free foods. J. Proteomics.

[bib7] Brouns J.P.H., van Buel V.J., Shewry P.R. (2013). Does wheat make us fat and sick?. J. Cereal. Sci..

[bib8] Carnevali A., Gianotti A., Benedett S., Tagliamonte M.C., Primiterra M., Laghi L., Danesi F., Valli V., Ndaghijimana M., Capozzi F., Canestrari F., Bordoni A. (2014). Role of Kamut® brand khorasan wheat in the counteraction of non-celiac wheat sensitivity and oxidative damage. Food Res. Int..

[bib9] Claus A., Schreiter P., Weber A., Graeff S., Herrmann W., Claupein W., Schieber A., Carle R. (2006). Influence of agronomic factors and extraction rate on the acrylamide contents in yeast-leavened breads. J. Agric. Food Chem..

[bib10] Colomba M.S., Gregorini A. (2012). Are ancient durum wheats less toxic to Celiac patients? A study of α-gliadin from Graziella Ra and Kamut. Sci. World J..

[bib11] Colomba M., Vischi M., Gregorini A. (2012). Molecular characterization and comparative analysis of six durum wheat accessions including Graziella Ra. Plant Mol. Biol. Rep..

[bib12] Colomba M.S., Gregorini A. (2011). Genetic diversity analysis of the durum wheat Graziella Ra, *Triticum turgidum* L. subsp. *durum* (Desf.) Husn. (Poales, Poaceae). Biodivers. J..

[bib13] De Vincenzi M., Luchetti R., Giovannini C., Pogna N.E., Saponaro C., Galterio G., Gasbarrini G. (1996). In vitro toxicity testing of alcohol-soluble proteins from diploid wheat *Triticum monococcum* in celiac disease. J. Biochem. Toxicol..

[bib14] De Vita P., Ficco D.B.M., Luciani A., Vincentini A., Pettoello-Mantovani M., Silano M., Msaiuri L., Cattivelli L. (2012). A ω-secalin decamer shows a coeliac disease prevention activity. J. Cereal. Sci..

[bib15] Dinu M., Whittaker A., Pagliai S., Sofi F. (2018). Ancient wheat species and human health: biochemical and clinical implications. J. Nutr. Biochem..

[bib16] Dubcovsky J., Dvorak J. (2007). Genome plasticity a key factor in the success of polyploidy wheat under domestication. Science.

[bib17] Feldman M., Bonjean A.P., Angus W.J. (2001). Origin of cultivated wheat. The World Wheat Book: a History of Wheat Breeding.

[bib18] Gazza L., Vincentini O., De Vincenzi M., Piccinini M., Petrangeli V., Ng P.K.W., Pogna R.E., Branlard G. (2010). Variation in toxicity of ‘monococcum’ and dicoccum’ wheat plants for celiac patients. Proceedings 10^th^ International Gluten Workshop.

[bib19] Gelinas P., McKinnon C. (2016). Gluten weight in ancient and modern wheat and the reactivity of epitopes towards R5 and G12 monoclonal antibodies. Int. J. Food Sci. Technol..

[bib20] Gell G., Kovács K., Molnár Z., Sándor T., Juhász A. (2015). Celiac disease-specific prolamin peptide content of wheat relatives and wild species determined by ELISA assays and bioinformatics analyses. Cereal Res. Commun..

[bib21] Gianfrani C., Camarca A., Mazzarella G., Di Stasio L., Giardullo N., Ferranti P., Picariello G., Rotondi Aufiero V., Picascia S., Troncone R., Pogna N., Auriccio S., Mamone G. (2015). Extensive *in vitro* gastrointestinal digestion markedly reduces the immune-toxicity of *Triticum monococcum* wheat: implication for celiac disease. Mol. Nutr. Food Res..

[bib22] Gianfrani C., Maglio M., Rotondi Aufiero V., Camarca A., Vocca I., Iaquinto G., Giardullo N., Pogna N., Troncone R., Auricchio S., Mazarella G. (2012). Immunogenicity of monococcum wheat in celiac patients. Am. J. Clin. Nutr..

[bib23] Gianotti A., Danesi F., Verardo V., Serrazaneti D.I., Valli V., Russo A., Riciputi Y., Tossani N., Caboni M.F., Guerzoni M.E., Bordoni A. (2011). Role of cereal type and processing in whole grain *in vivo* protection from oxidative stress. Front. Biosci..

[bib24] Godfrey D., Hawkesford M., Powers S., Millar S., Shewry P.R. (2010). Nutritional effects on wheat grain composition and end use quality. J. Agric. Food Chem..

[bib25] Granvogel M., Wiester H., Koehler P., von Tucher S., Schieberle P. (2007). Influence of sulfur fertilization on the amounts of free amino acids in wheat. Correlation with baking properties as well as with 3-aminopropionamide and acrylamide generation during baking. J. Agric. Food Chem..

[bib26] Gregorini A., Coloma M., Ellis H.J., Ciclitira P.J. (2009). Immunogenicity characterization of two ancient wheat α-gliadin peptides related to coeliac disease. Nutrients.

[bib27] Hawkesford M.J., Zhao F.-J. (2017). Strategies for increasing the selenium content of wheat. J. Cereal. Sci..

[bib28] Iacomino G., Di Stasio L., Fierro O., Picariello G., Venezia A., Gazza L., Ferranti P., Mamone G. (2016). Protective effects of ID331 *Triticum monococcum* gliadin on *in vitro* models of the intestinal epithelium. Food Chem..

[bib29] Klockenbring T., Boese A., Bauer R., Goerlich R. (2001). Comparative investigations of wheat and spelt cultivars: IgA, IgE, IgG1 and IgG4 binding characteristics. Food Agric. Immunol..

[bib30] Marotti I., Bregola V., Aloisio I., Di Gioia D., Bois S., Di Silvestro R., Quinn, Dinelli G. (2011). Prebiotic effect of soluble fibres from modern and old durum-type wheat varieties on *Lactobacillus* and *Bifidobacterium* strains. J. Sci. Food Agric..

[bib31] Mattei J., Malik V., Wedick N.M., Hu F.B., Spiegelman D., Willett W.C., Campos H., Global Nutrition Epidemiologic Transition Initiative (2015). Reducing the global burden of type 2 diabetes by improving the quality of staple foods: the Global Nutrition and Epidemiologic Transition Initiative. Glob. Health.

[bib32] Molberg O., Uhlen A.K., Jensen T., Flaete N.S., Fleckenstein B., Arentz-Hansen H., Raki M., Lundin K.E.A., Sollid L.M. (2005). Mapping of gluten T-cell epitopes in the bread wheat ancestors: implications for celiac disease. J. Gastroenterol..

[bib33] Morris C.E., Sands D.C. (2003). The breeder's dilemma – yield or nutrition?. Nat. Biotechnol..

[bib34] Moshenska G. (2017). Esoteric egyptology, seed science and the mmyth of mummy wheat. Open Libr. Humanit..

[bib35] NDND 2014. National Diet and Nutrition Survey Results from Years 1, 2, 3 and 4 (combined) of the Rolling Programme (2008/2009 – 2011/2012) Public Health England. 160pp. https://www.gov.uk/government/statistics/national-diet-and-nutrition-survey-results-from-years-1-to-4-combined-of-the-rolling-programme-for-2008-and-2009-to-2011-and-2012.

[bib36] Pizzuti D., Buda A., D'Odorico A., D'Inca R., Chiarelli S., Curioni A., Martines D. (2006). Lack of intestinal mucosal toxicity of *Triticum monococcum* in celiac disease patients. Scand. J. Gastroenterol..

[bib37] Quinn R.M., Janick J. (1999). Kamut®: ancient grain, new cereal. Perspective on New Crops and New Uses.

[bib38] Ribeiro M., Rodroguez-Quijano M., Nunes F.M., Carillo J.M., Branlard G., Igrejas G. (2016). New insights into wheat toxicity: breeding does not seem to contribute to a prevalence of potential celiac disease's immunostimulatory epitopes. Food Chem..

[bib39] Righetti L., Rubert J., Galavena G., Folloni S., Ranieri R., Stranska-Zachariasova M., Hajslova J., Dall'Asta C. (2016). Characterisation and discriminant of ancient grains: a metabolomics approach. Int. J. Mol. Sci..

[bib40] Saa D.T., Turroni S., Serrazanetti D.I., Rampelli S., Maccaferri S., Candela M., Severgnini M., Simonetti E., Brigidi P., Gianotti A. (2014). Impact of Kamut® Khorasan on gut microbiota and metabolome in healthy volunteers. Food Res. Int..

[bib41] Sapone A., Bai J.C., Ciacci C., Dolinsek J., Green P.H.R., Hadjivassiliou M., Kaukinen K., Rostami K., Sanders D.S., Schumann M., Ullrich R., Villalta D., Volta U., Catassi C., Fasano E. (2012). Spectrum of gluten-related disorders: consensus on new nomenclature and classification. BMC Med..

[bib42] Scazzina F., Del Rio D., Serventi L., Carini E., Vittadini E. (2008). Development of nutritionally enhanced tortillas. Food Biophys..

[bib43] Shewry P.R. (2007). Improving the protein content and composition of cereal grain. J. Cereal. Sci..

[bib44] Shewry P.R., Hey S. (2015). The contribution of wheat to human diet and health. Food and Energy Security.

[bib45] Shewry P.R., Hey S. (2015). Do “ancient” wheat species differ from modern bread wheat in their contents of bioactive components?. J. Cereal. Sci..

[bib46] Shewry P.R., Hey S. (2016). Do we need to worry about eating wheat?. Nutr. Bull..

[bib47] Shewry P.R., Gebruers K., Andersson A.A.M., Aman P., Piironen V., Lampi A.-M., Boros D., Rakszegi M., Bedo Z., Ward J.L. (2011). Relationship between the contents of bioactive components in grain and the release dates of wheat lines in the healthgrain diversity screen. J. Agric. Food Chem..

[bib48] Shewry P.R., Piironen V., Lampi A.-M., Edelmann M., Kariluoto S., Nurmi T., Fernandez-Orozco R., Ravel C., Charmet G., Andersson A.A.M., Åman P., Boros D., Gebruers K., Dornez E., Courtin C.M., Delcour J.A., Rakszegi M., Bedő Z., Ward J.L. (2010). The HEALTHGRAIN wheat diversity screen: effects of genotype and environment on phytochemicals and dietary fiber components. J. Agric. Food Chem..

[bib49] Shewry P.R., Hawkesford M.J., Piironen V., Lampi A.-M., Gebruers K., Boros D., Anderson A.A.M., Aaman P., Rakzegi M., Bedo Z., Ward J.L. (2013). Natural variation in grain composition of wheat and related cereals. J. Agric. Food Chem..

[bib50] Shewry P.R., Pellny T., Lovegrove A. (2016). Is modern wheat bad for health?. Nat. Plants.

[bib51] Shewry P.R., Coral D.I., Jones H.D., Beale M., Ward J. (2017). Defining genetic and chemical diversity in wheat grain by 1H-NMR spectroscopy of polar metabolites. Mol. Nutr. Food Res..

[bib52] Simonato G.P., Giannattasio M., Curioni A. (2002). Allergic potential of Kamut® wheat. Allergy.

[bib53] Sofi F., Whittaker A., Cesari F., Gor A.M., Becatti M., Dinelli G., Casini A., Abbate R., Gensini G.F., Benedettelli S. (2013). Characterization of Khorasan wheat (Kamut) and impact of a replacement diet on cardiovascular risk factors: a cross over dietary intervention study. Eur. J. Clin. Nutr..

[bib54] Sofi F., Whittaker A., Gori A.M., Cesri F., Surrenti E., Abbate R., Gensini G.F., Benedettelli S., Casini A. (2014). Effect of *Triticum turgidum* subsp. turanicum wheat on irritable bowel syndrome: a double-blinded randomised dietary intervention trial. Br. J. Nutr..

[bib55] Sollid L.M., Qiao S.-W., Anderson R.P., Gianfrani C., Koning F. (2012). Nomenclature and listing of celiac disease relevant gluten T-cell epitopes restricted by HLA-DQ molecules. Immunogenetics.

[bib56] Spaenij-Dekking L., Kooy-Winkelaar Y., Van Veelan P., Wouter Drijfhout J., Jonker H., Van Soest L., Smulders M.J.M., Bosch D., Gilissen L.J.W.J., Koning F. (2005). Natural variation in toxicity of wheat: potential for selection of nontoxic varieties for celiac disease patients. Gastroenterology.

[bib57] Šuligoj T., Gregorini A., Colomba M., Ellis H.J., Ciclitira P.J. (2013). Evaluation of the safety of ancient strains of wheat in coeliac disease reveals heterogenous small intestinal T cell responses suggestive of coeliac toxicity. Clin. Nutr..

[bib58] Valerii M.C., Ricci C., Spisni E., Di Silvestro R., De Fazio L., Cavazza E., Lanzini A., Campierei M., Dalpiaz A., Pavan B., Volta U., Dinelli G. (2015). Responses of peripheral blood mononucleated cells from non-celiac gluten sensitive patients to various cereal sources. Food Chem..

[bib59] Valli V., Danesi F., Gianotti A., Di Nunzio M., Saa D.T., Bordoni A. (2016). Antioxidative and anti-inflammatory effect of *in vitro* digested cookies baked using different types of flours and fermentation methods. Food Res. Int..

[bib60] van den Broeck H., Honbing C., Lacaze X., Dusautoir J.-C., Gilissen L., Smulder M., van der Meer I. (2010). In search of tetraploid wheat accessions reduced in celiac disease-related gluten epitopes. Mol. Biosyst..

[bib61] van den Broeck H.C., de Jong H.C., Salentijn E.M.J., Dekking L., Bosch D., Hamer R.J., Gilissen L.J.W.J., van der Meer I.M., Smulders M.J.M. (2010). Presence of celiac disease epitopes in modern and old hexaploid wheat varieties: wheat breeding may have contributed to increased prevalence of celiac disease. Theor. Appl. Genet..

[bib62] Vincentini O., Borrelli O., Silano M., Gazza L., Pogna N., Luchetti R., De Vincenzi M. (2009). T-cell response to different cultivars of farro wheat, *Triticum turgidum* ssp. *Dicoccum*, in celiac disease patients. Clin. Nutr..

[bib63] Vincentini O., Maialetti F., Gazza L., Silano M., Dessi M., De Vincenzi M., Pogna N.E. (2006). Environmental factors of celiac disease: cytotoxicity of hulled wheat species Triticum monococcum, *T. turgidum* ssp. dicoccum and *T. aestivum* ssp. spelta. J. Gastroenterol. Hepatol..

[bib64] Vu N.T., Pasco J.A., Kovács A., Wing L.W., Békés F., Suter D.A.I. (2014). The prevalence of wheat and spelt sensitivity in a randomly selected Australian population. Cereal Res. Commun..

[bib65] Ward J.L., Poutanen K., Gebruers K., Piironen V., Lampi A.-M., Nyström L., Andersson A.A.M., Åman P., Boros D., Rakszegi R., Bedő Z., Shewry P.R. (2008). The HEALTHGRAIN cereal diversity screen: concept, results and prospects. J. Agric. Food Chem..

[bib66] Whittaker A., Sofi F., Luisi M.L.E., Rafanelli E., Fiorello C., Becatti M., Abbate R., Casini A., Gensini G.F., Benedettelli S. (2015). An organic Khorasan wheat-based replacement diet improves risk profile of patients with acute coronary syndrome: a randomized crossover trial. Nutrients.

[bib67] Whittaker A., Dinu M., Cesari F., Gori A.M., Fiorello C., Becatti M., Casini A., Marcucci R., Benedettelli S., Sofi F. (2017). A khorasan wheat-based replacement diet improves the risk profile of patients with type 2 diabetes mellitus (T2DM): a randomized crossover trial. Eur. J. Nutr..

[bib68] Ziegler J.U., Steingass C.B., Longin C.F.H., Wurschum T., Carle R., Schweiggert R.M. (2015). Alkylresorcinol composition allows the differentiation of *Triticum* spp. having different levels of ploidy. J. Cereal. Sci..

[bib69] Ziegler J.U., Steiner D., Longin C.F.H., Wurschum R.M., Schwiggert R.M., Carle R. (2016). Wheat and the irritable bowel syndrome- FODMAP levels of modern and ancient species and their retention during bread making. J. Funct. Foods.

[bib70] Zuidmeer L., Goldhahn K., Rona R.J., Gislason D., Madsen C., Summers C., Sodergren E., Dahlstrom J., Lindner T., Sigurdardottir S.T., McBride D., Keil T. (2008). The prevalence of plant food allergies: a systematic review. J. Allergy Clin. Immunol..

